# Qualitative evaluation of Thailand’s National Tobacco Control Strategy 2022–2027: Challenges and suggestions for policy implementation

**DOI:** 10.18332/tid/203935

**Published:** 2025-05-10

**Authors:** Chakkraphan Phetphum, Artittaya Wangwonsin, Orawan Keeratisiroj, Wutthichai Jariya

**Affiliations:** 1 Department of Community Health, Faculty of Public Health, Naresuan University, Phitsanulok, Thailand; 2 Tobacco Control Research Unit, Naresuan University, Phitsanulok, Thailand

**Keywords:** Thailand, WHO FCTC, policy evaluation, policy implementation, tobacco control policies

## Abstract

**INTRODUCTION:**

The WHO Framework Convention on Tobacco Control has been aligned with Thailand's National Tobacco Control Strategy (NTCS), which has been implemented for over three decades. However, policy evaluation is essential to improve its effectiveness. This formative evaluation study aims to identify challenges and provide suggestions for implementing the current NTCS 2022–2027 at both national and operational levels.

**METHODS:**

Data collection for this qualitative research involved document reviews and in-depth interviews using a semi-structured interview guide. Thematic analysis was used to identify and analyze data from 15 informants who were purposively selected based on their influential positions, expertise, and experience in NTCS implementation.

**RESULTS:**

Thailand's NTCS operates through both national and operational stakeholders. The findings revealed multifaceted challenges and suggestions across these levels, categorized into six key areas. First, the gaps in strategic coordination and prioritization reinforced the need to strengthen intersectoral partnerships to achieve tobacco control as a national priority. Second, problems with monitoring as well as evaluation and reporting processes highlighted the need for clearly defined key performance indicators and timelines. Third, role clarity-related gaps at the ‘tobacco control focal point’ revealed a need for capacity building and strategic management support. Fourth, policy dissemination was impeded by one-way communication modes, emphasizing the importance of two-way communication to foster stakeholder engagement. Fifth, centralized budget management hindered ready matching of resources with regional needs, urging the implementation of decentralized management. Finally, the lack of innovation in tobacco control challenges demonstrated the need for knowledge-sharing mechanisms.

**CONCLUSIONS:**

The evaluation offers important insights to strengthen NTCS implementation through enhanced coordination, systematic monitoring, capacity development, policy dissemination, allocation of adequate funds, and promoting innovations. These results contribute to the evidence base for effective tobacco control strategy by providing tangible recommendations for policymakers.

## INTRODUCTION

Tobacco is a major cause of disease burden, resulting in about 6 million deaths annually worldwide^1^. Tobacco use and tobacco-related deaths are disproportionately high in low- and middle-income countries, including Thailand^2^. More than 14.6 million individuals in Thailand consume tobacco, resulting in >80000 deaths annually and accounting for 18% of all fatalities^3^. According to the World Health Organization (WHO) global report on trends in prevalence of tobacco use, the prevalence of daily smoking among Thai adults decreased from 24% in 2011 to 21% in 2016^4^. More recently, the WHO Report on the Global Tobacco Epidemic 2023 indicates that approximately 16% of Thai adults are current daily smokers, reflecting a continued downward trend^5^. Additionally, the estimated social costs of tobacco consumption in Thailand amounted to US$2.18 billion, which accounted for approximately 18.19% of total health expenditures and 0.78% of the GDP. The tobacco industry contributed only 0.50% of the total GDP^6^. In order to mitigate the burdens associated with tobacco use, intensive efforts have significantly expanded and reinforced tobacco control activities by using a variety of effective interventions and policies^5^.

The WHO Framework Convention on Tobacco Control (FCTC) offers countries a comprehensive policy framework to implement tobacco control programs^7,8^. Thailand has announced the National Tobacco Control Strategy (NTCS) in accordance with the FCTC for the past three decades with the participation and cooperation of all sectors, including government, non-government organizations, private sector, and civil society^9-12^. This initiative began in 2010 with the first edition of NTCS covering the years 2010–2014, followed by the second edition covering the years 2016–2019. The NTCS 2016–2019 played a critical role in strengthening multi-sectoral collaboration, enhancing enforcement of smoke-free laws, and promoting cessation services, reducing tobacco use, and improving coordination across agencies. However, the challenges remain, such as inconsistent local-level implementation and limited data use in planning. These experiences informed the development of the third edition of NTCS, spanning from 2022–2027, which seeks to build on previous efforts while addressing existing gaps^9-12^.

Currently, Thailand is in the process of implementing the NTCS 2022–2027. It consists of six strategies: consumption regulation, enhance public awareness of the harmful effects of smoking, assistance for those who want to quit smoking, disclosure of tobacco product contents, smoking bans in public places, tobacco tax reform and a crackdown on illegal tobacco sales^9-12^. These third editions of the NTCS share two common objectives: to reduce the prevalence of smoking among the population and to protect public health from the dangers of cigarette smoke. Achieving these objectives as outlined will help decrease the incidence of chronic non-communicable diseases, reduce the loss of healthy years of life, and lessen the economic burden on the country^9-12^.

Evaluating the tobacco control policies during and after their implementation is a vital part of the policy-making process^13,14^. The analysis of the country’s strategic policy for tobacco control has identified shared challenges across Bangladesh, Ethiopia, India and Uganda, such as the level of understanding is limited beyond health agencies, conflicting mandates, and obstacles to vertical coordination^15^. In India, the state level is pivotal in the adoption and execution of policies. States must provide a favorable politico-administrative framework and allocate institutional structures and resources to implement concrete initiatives. They must prioritize the development of a sub-national policy framework, accompanied by requisite resources, institutions, and capabilities^16^. Key agencies of Kenya’s tobacco control policy comprise governmental commitment, stakeholder engagement, and financial support. Challenges include industry intervention, inadequate enforcement, and unclear roles, requiring enhanced resources and stakeholder engagement for effective implementation^17^. The challenges to enforcing the National Tobacco Control Act in Nigeria include poorly designed health warnings, low tobacco taxation, and a deficiency in regulatory independence about the Act. Improvements to the revised warnings and their implementation are advised^18^.

In Thailand, the evaluation of national strategic policy for tobacco control reveals significant implications and challenges^19,20^. The previous study highlighted that, while the accomplishments in tobacco control in Thailand have fostered a sense of achievement within Thai society and government, there is a need to prioritize the development of a national action plan for tobacco control. This requires the participation of many stakeholders and the mapping of strategies and activities towards a clearly defined national tobacco control program^19^. Another study evaluated the challenges of Thailand which has four policies and laws concerning tobacco-related issues. It emerged that Thailand’s policies on tobacco control are strong and consonant with FCTC concerning taxation, media advertising and warning labels. Some areas of challenges were roll-your-own cigarettes, selling to under-aged people, household smoking, illicit trade, support for tobacco growers, and liability. Addressing these can further reduce tobacco consumption^20^.

Two studies evaluated the effectiveness and situation of the first edition of NTCS 2010–2014^21,22^. The first study analyzed collected data in relation to the plan’s objectives and recommended incorporating smoking cessation programs into universal healthcare, adding quit-smoking pharmaceuticals to essential medicines lists, and prioritizing tobacco control initiatives at the local level^21^. The second study provided seven suggestions: implementing tobacco control policy and leadership training, establishing organizational structures and management systems, creating surveillance, monitoring, and evaluation systems, supporting research and knowledge management strategies, enhancing capacity and expanding networks for tobacco control across sectors, strengthening collaborative networks for tobacco control at regional levels, and enhancing and enforcing tobacco control laws^22^.

Based on the literature review in Thailand, there has been limited evaluation of the tobacco-related policies and laws^19,20^ and the initial edition of NTCS 2010–2014^21,22^. Furthermore, there remains a dearth of research on the evaluation of the second edition of NTCS 2016–2019 and the third edition of NTCS 2022–2027. Therefore, this study applied the formative evaluation to evaluate the early-stage implementation of the current NTCS 2022–2027. The main objectives of this study were to identify the challenges and provide suggestions for policy implementation at the national and operational levels. The findings can support timely and responsive policy adjustments, strengthen coordination across sectors, and enhance the effectiveness of implementation mechanisms. Additionally, the study provides practical recommendations that may guide strategic planning, resource management, and institutional accountability during the remaining years of the NTCS. These insights are valuable not only for improving the current strategy but also for informing future tobacco control efforts in Thailand.

## METHODS

This study employed a ‘reality-oriented’ qualitative inquiry approach, which believes that informants’ perspectives are shaped by and grounded in their real-world experiences^23^. This approach was appropriate for exploring the actual implementation practices, challenges, and opportunities related to the NTCS. It enabled the researchers to capture and conceptualize how policy is executed in real-world settings, based on the perspectives of those directly involved in the implementation process. As a result, the findings of this study are highly relevant and benefit to policymakers seeking to make realistic and timely adjustments to policy implementation strategies. In addition, as part of a formative evaluation^14,23^, the unit of analysis was the implementation process of the NTCS 2022–2027 at both the national and operational levels, including regional and provincial contexts^8^. This study was conducted and presented in accordance with the standards for reporting qualitative research^24^ (Supplementary file).

### Ethical issues

This study complied with ethical standards and was approved by the Institutional Review Board (Certification No. COA No.085/2023, IRB No. P3-0024/2023; certified on 11 April 2023). The study adhered to the principles outlined in the Declaration of Helsinki. No financial support was acquired from tobacco-related enterprises. Prior to the interviews, informants received an information sheet detailing the study and were asked to sign informed consent forms. These forms highlighted the confidentiality of their responses, the voluntary nature of participation, and their right to withdraw from the study at any point without justification. To reduce potential social desirability and response bias, informants were reassured that their responses would remain anonymous and would not affect their roles or relationships with any organization. All identifying information was removed during transcription, and informants were assigned anonymous codes in the presentation of results to ensure confidentiality.

### Data collection

Data were collected by the research team which collectively possesses a wealth of experience and knowledge in the fields of public health policy, qualitative research methodologies, and tobacco control policy, and each member has contributed their unique expertise to this study.

Document reviews and in-depth interviews were utilized to collect data^23^. During the document review phase between July and September 2023, we systematically reviewed a broad range of literature and policy documents to capture the evolution, structure, and implementation mechanisms of Thailand’s NTCS^9-12,19-22,25^. Main sources included: 1) legislative frameworks such as the Tobacco Products Control Act B.E. 2560 (2017) and related ministerial regulations; 2) government-issued reports and policy documents including the National Tobacco Control Strategy 2022–2027 and its prior editions; 3) implementation and progress reports from the Department of Disease Control and the Bureau of Tobacco Control; 4) circulars and official orders disseminated to relevant ministries and agencies; and 5) peer-reviewed journal articles and academic reports on Thailand’s tobacco control efforts. This systematic methodology enabled a comprehensive understanding of the NTCS’s evolution from its origin to its present third edition, including its implementation structure and mechanisms. Thus, we meticulously identified key informants for the forthcoming in-depth interviews based on the insights gained from the document analysis.

During the in-depth interview phase between October 2023 and March 2024, three researchers (CP, AW, WJ) conducted and facilitated the interviews during the in-depth interview phase, while another researcher (OK) served as the note-taker. The use of a consistent interview team helped maintain methodological uniformity and reduce interviewer bias. The informants were intentionally and voluntarily chosen based on their influential positions, expertise, and experience in the NTCS implementation, thereby guaranteeing the acquisition of comprehensive and rich data^23^. A total of 15 informants (coded as I1–I15) were interviewed, with 19 sessions, including repeat interviews with four informants (I1, I5, I11, and I13) to obtain additional information (Table 1).

**Table 1:** Characteristics and roles of key informants involved in NTCS implementation (N=15)

*Informants*	*Representative of* *informants’ level*	*Affiliation*	*Position in NTCS* *implementation*	*Years of experience* *in NTCS implementation*
I1	National	Representative of academic sector	Committee for NCTC	10
I2	National	Tobacco Control Research and Knowledge Management Center	Committee for NCTC	5
I3	National	Representative of private sector	Committee for NCTC	40
I4	National	Ministry of Public Health	Secretariat for NCTC	10
I5	National	Department of Disease Control	Secretariat for SC-NTCS	2
I6	National	Department of Disease Control	Secretariat for SC-NTCS	1
I7	National	Department of Disease Control	Secretariat for SC-NTCS	1
I8	National	Department of Disease Control	Secretariat for SC-NTCS	1
I9	National	Department of Disease Control	Secretariat for SC-NTCS	1
I10	National	Department of Disease Control	Secretariat for SC-NTCS	1
I11	Operational	Office of Disease Prevention and Control	Implementor for NTCS	10
I12	Operational	Office of Disease Prevention and Control	Implementor for NTCS	5
I13	Operational	Provincial Public Health Office	Implementor for NTCS	10
I14	Operational	Provincial Public Health Office	Implementor for NTCS	3
I15	Operational	Provincial Public Health Office	Implementor for NTCS	4

NCTC: National Committee for Tobacco Control. NTCS: National Tobacco Control Strategy. SC-NTCS: Steering Committee for National Tobacco Control Strategy.

In-depth interviews were conducted using a semi-structured interview guide, a method that is widely recognized for its ability to elicit detailed and abundant qualitative data^23^. The guide was created in accordance with the study’s objectives. To ensure content validity and clarity, the draft guide was reviewed by two qualitative research experts. It was then pre-tested with two informants (not included in the final analysis), and minor revisions were made based on their feedback prior to data collection. The guide had open-ended questions addressing essential areas, including experiences and perspectives of NTCS implementation, encountered problems, and suggestions for development.

During the interview sessions, the research team established rapport with each informant by introducing themselves and clearly explaining the study’s aims. This facilitated a comfortable environment for open dialogue^23^. We prompted informants with probing inquiries to articulate their opinions and insights openly, while avoiding leading or suggestive language^23^. These procedures were designed to minimize potential response bias by ensuring that informants could express their perspectives freely and authentically. In addition, if informants had documents related to NTCS implementation, they were invited to share them to support the research team’s understanding and enhance the depth and contextual richness of the data collected.

Interview sessions were arranged at times and venues that were jointly agreed upon by both the informants and the research team, ensuring convenience and accessibility for all parties involved. Eleven interviews were conducted remotely through Zoom Meetings, while the remaining four took place on-site at the informants’ workplaces. Four repeated interviews were conducted by phone. Each interview session continued until data saturation was reached, with durations ranging from 40 to 110 minutes. The informants were requested to grant permission for audio recording and verbatim transcription. This ensured that all informants’ perspectives were captured in full, thereby reducing the risk of interpretation bias and ensuring analytic rigor.

### Data analysis

Thematic analysis was employed to identify and analyze patterns of meaning or themes within the data expressed by informants^26^. Our research team, composed of four members, collectively analyzed the data based on the data-driven approach^26^. After each interview session, we familiarized ourselves with the data by repeated reading of the transcripts and then independently conducting line-by-line coding. The initial codes were then organized into potential themes. Subsequently, collaborative meetings were arranged among the research team to compare codes and potential themes, ultimately finalizing, and naming the themes^26^. We employed Microsoft Excel (Microsoft Corporation, Redmond, WA) to organize the data and arrange the emerging themes, as it was readily accessible to our research team and offered a cost-effective approach for managing qualitative data^27^. During the meeting, we discussed both the similarities and differences in data interpretation. Through investigator triangulation, the research team contributed diverse perspectives and insights, helping to mitigate individual interpretation bias^28^. Furthermore, the analyzed findings were shared with informants for verification (member checking) to reduce researcher bias and enhance the trustworthiness of the data analysis and overall study results^28^.

## RESULTS

**Figure 1:**
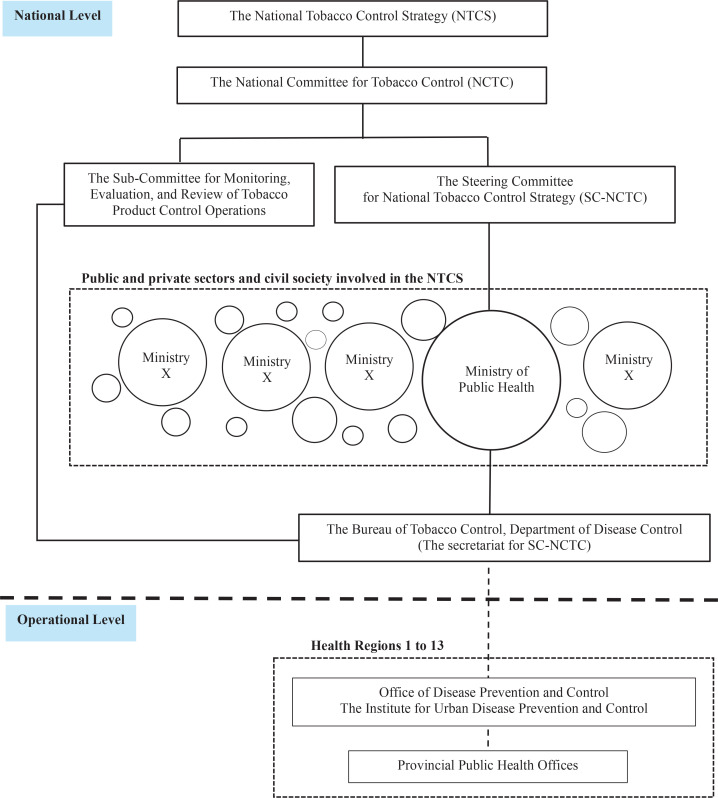
Implementation structure and mechanisms of Thailand’s National Tobacco Control Strategy

The findings from the document review illustrate that the National Tobacco Control Strategy (NTCS) in Thailand operates through a multi-level framework involving national and operational stakeholders, as shown in Figure 1. At the national level, the National Committee for Tobacco Control (NCTC) oversees the strategy, providing management, supervision, and recommendations. The Steering Committee for the National Tobacco Control Strategy (SC-NTCS) coordinates six principal strategies: strengthening tobacco control capacity, preventing new tobacco users, supporting cessation efforts, regulating tobacco products, promoting smoke-free environments, and enforcing taxation and other regulatory measures. These strategies are developed and monitored through collaboration among representatives from ministries such as Public Health, Interior, Finance, and Education. The Bureau of Tobacco Control, under the Department of Disease Control, serves as the secretariat for the SC-NTCS, facilitating inter-agency collaboration, coordinating meetings, and consolidating operational data for reporting. Moreover, it gathers operational data from relevant agencies regarding the performance indicators of each plan and compiles reports to be submitted to the Sub-Committee for Monitoring, Evaluation, and Review of Tobacco Product Control Operations.

At the operational level (Figure 1), national policies and performance indicators are communicated through state-level agencies to regional and local units. For instance, the Ministry of Education transmits policies to district offices and schools, while the Ministry of Interior and the Ministry of Finance do so to local administrative and revenue offices, respectively. The Bureau of Tobacco Control translates strategies into actionable plans, distributes resources, and monitors Provincial Public Health Offices (PHOs), which develop activities aligned with NTCS objectives.

The Provincial Public Health Offices operate within 13 health regions (Figure 1). Health Regions 1 to 12 are supported by the Office of Disease Prevention and Control which oversees budgeting, supervision, monitoring, and consultation. The Metropolitan Health Region (Health Region 13) in Bangkok is administered by the Institute for Urban Disease Prevention and Control instead of the Office of Disease Prevention and Control. The Office of Disease Prevention and Control and the Institute for Urban Disease Prevention and Control are essential in ensuring provincial compliance with the NTCS by overseeing and assisting the Provincial Public Health Offices in the effective execution of tobacco control initiatives.

The findings from the in-depth interviews are presented in two sections. The first section explores the challenges identified, and the second section provides suggestions for improvement. Emerging themes are synthesized and presented in Table 2.

**Table 2:** Challenges and suggestions classified by levels of policy implementation, derived from thematic analysis of in-depth interview data

*Levels*	*Challenges*	*Suggestions*
**National level**	Gaps in strategic coordination and prioritization	Strengthen partnerships among strategy-driving committees
	Organizational and role clarity deficiencies of secretariats for SC-NTCS	Develop strategic support systems for secretariat for SC-NTCS
	Weaknesses in indicator monitoring and reporting systems	Improve monitoring and evaluation frameworks
	Lack of effective strategic plan dissemination and engagement	Enhance policy communication strategies to foster engagement and understanding
**Operational level**	Centralized budget management	Develop budget allocation strategies based on local needs
	Limited prioritization of tobacco control by agency leaders	Strengthen engagement of government and civil society
	Insufficient data for effective planning and evaluation	Develop a comprehensive database system for planning and evaluation
	Limited innovation in addressing tobacco control challenges	Facilitate technology and knowledge sharing on successful innovations

SC-NTCS: Steering Committee for National Tobacco Control Strategy.

### Challenges of policy implementation

*Gaps in strategic coordination and prioritization*

Tobacco control efforts face significant challenges due to limited collaborative planning and indicator monitoring across ministries. Without a cohesive framework, it is difficult to align strategies and measure progress effectively:

*‘We haven’t really sat down and talked enough about what this strategic plan should do and where we’re heading. Everyone’s just doing their own thing, with no joint planning or concrete reports to assess progress.’* (I4)

Strategic steering organizations often give low priority to tobacco control, which results in indicators being treated as non-urgent and diminishes attention to tobacco-related issues:

*‘Ministry executives don’t see tobacco control as a priority. Some ministries cooperate well, but others show limited engagement.’* (I5)

This low prioritization is further compounded by frequent personnel turnover, which disrupts continuity and weakens coordination:

*‘Representatives rotate frequently, so it’s not the same person consistently participating, resulting in discontinuity in operations.’* (I9)

*Organizational and role clarity deficiencies of secretariat for the SC-NTCS*

The Bureau of Tobacco Control appointed division heads as secretaries for each NTCS strategy, assigning them responsibilities such as preparing agendas, monitoring progress, and reporting issues to chairpersons. However, many secretaries struggle to fulfill these duties due to unclear role definitions:

*‘The secretaries assigned to each strategy may not fully understand their responsibilities – what needs to be done, how to oversee and monitor the plans, or how to present issues to the chairpersons. As a result, many strategies have less progress toward tangible outcomes.’* (I6)

This challenge is caused by frequent personnel turnover, insufficient information transfer, and the absence of a central coordinating body:

*‘The turnover rate for secretaries is high, which leads to new personnel not understanding their roles. Those leaving the position often do not hand over information. Each strategy's secretary tends to work in isolation because there is no central coordinator for overseeing the entire strategy.’* (I10)

*Weaknesses in indicator monitoring and reporting systems*

A major challenge in implementing the NTCS is the absence of clear and measurable indicators for tracking progress. Many indicators are vaguely defined, without target benchmarks, and do not include evaluation methods. This makes it difficult to assess success:

*‘We encounter issues with indicators that unclear measurable targets. This affects our ability to determine at what level success can be declared. For instance, the indicator 80% of the population has knowledge and awareness of toxic substances in tobacco products’ fails to specify the target population or evaluation criteria.’* (I1)

In addition, uncoordinated reporting systems across organizations lead to inconsistencies and limit data quality:

*‘The largest database we rely on is the Health Data Center of the Ministry of Public Health. However, other data sources, such as local government systems or private software used by some hospitals, are not integrated. This fragmentation prevents us from consolidating nationwide data into a single system.’* (I7)

*Lack of effective strategic plan dissemination and engagement*

The dissemination of the strategic plan relied heavily on one-way communication, such as official letters and policy documents sent to operational-level agencies. In most cases, dissemination occurs through policy briefings or presentations delivered by invited speakers during meetings. This passive approach led to limited understanding of the plan’s strategies and indicators. It failed to foster a sense of shared responsibility among stakeholders:

*‘We (the Bureau of Tobacco Control) created the strategic plan and shared it with agencies via official letters and meetings. Unfortunately, it often got ignored, just filed away. Agencies didn't really understand their roles or responsibilities, and they lacked a sense of ownership.’* (I5)

*Centralized budget management*

The centralized budget system overlooks local program demands and context-specific priorities. The Bureau of Tobacco Control distributes funding to 76 Provincial Public Health Offices based on the number of districts, without considering each province’s specific tobacco control challenges:

*‘The way funding is allocated based on the number of districts doesn't match actual needs. Some provinces with rising smoking rates get too little money, while others get more than needed, and the excess funds are returned to central authorities.’* (I13)

Budget cuts have further affected operations, especially within the Office of Disease Prevention and Control and the Provincial Public Health Offices:

*‘The Office of Disease Prevention and Control used to receive budgets in the millions, but last year it dropped to just hundreds of thousands.’* (I11)

These reductions made it impossible to hire project coordinators, leaving provincial staff overburdened:

*‘With no budget to hire coordinators, the workload at the provincial level has become even heavier. It's not just paperwork – we must carry out fieldwork and activities and coordinate with various agencies.’* (I14)

*Limited prioritization of tobacco control by agent leaders*

The success of NTCS implementation at the operational level depends heavily on the leadership of provincial governors and public health officers, who serve as chairpersons and secretaries of the Provincial Tobacco Control Committees. Their level of commitment directly influences the momentum of local tobacco control initiatives:

*‘When the leadership, like the governor or the provincial public health officer, prioritizes tobacco control as a key provincial agenda, it creates a strong push, encouraging various agencies to collaborate. Without this prioritization, tobacco control becomes just another routine task.’* (I10)

Insufficient leadership commitment often results in low morale and weak engagement among committee members:

*‘I've attended meetings at the provincial level where tobacco control indicators weren’t even discussed. Leaders only asked what agencies were doing in general. When leaders don’t take it seriously, committee members from different agencies also lose interest – they have no motivation to engage.’* (I15)

*Insufficient data for effective planning and evaluation*

Inadequate access to local-level data is a key challenge in implementing the NTCS and affects effective planning and evaluation:

*‘Although the Office of Disease Prevention and Control and Provincial Public Health Offices use survey data from the National Statistical Office, which conducts tobacco control surveys every three years, the absence of annual data limits planning and evaluation.’* (I12)

Without regular access to provincial, district, and sub-district data, agencies struggle to assess the scale and urgency of tobacco-related problems in specific areas. This limitation prevents them from identifying key determinants and understanding the potential impact on local populations.

*Limited innovation in addressing tobacco control challenges*

A major limitation in provincial tobacco control efforts is the minimal adoption of innovative strategies to respond to emerging challenges. Many projects continue to replicate familiar activities without adapting to changing contexts:

*‘Provincial projects are mostly repetitive, with no groundbreaking innovations. Activities still focus on traditional methods, like educating schoolchildren, placing stickers in public places, or directing people to smoking cessation clinics – which few use. If we could have new innovations, it could lead to more impactful solutions.’* (I15)

### Suggestions for policy implementation

*Strengthen partnerships among strategy-driving committees*

Strengthening partnerships among strategy-driving committees is essential to improve the implementation of tobacco control policies at the national level. Current efforts are hindered by limited collaboration and low prioritization from key ministries. The National Committee for Tobacco Control should adopt a whole-of-government approach because it helps to clarify roles and to ensure shared accountability:

*‘If we look at the FCTC, it’s a government mandate, not just the Ministry of Public Health's. However, other ministries often view it solely as the MOPH's responsibility. It's essential to clarify the roles of key ministries – like Interior, Education, Finance, Defense and others – and ensure they understand their mission to jointly support tobacco control efforts.’* (I13)

Establishing model organizations that integrate tobacco control into their performance systems can serve as powerful examples for others. The Ministry of Interior, for instance, included smoke-free indicators in its local performance assessments – leading to more than 6000 local administrative organizations meeting the criteria:

*‘Sharing such success stories could motivate other ministries to develop strategies aligned with national tobacco control goals.’* (I7)

*Develop strategic support systems for secretariats for the SC-NTCS*

To address role ambiguity and strengthen implementation, the Bureau of Tobacco Control should develop a strategic support system for the secretariats of the six NTCS strategies. One recommended approach is to appoint the Strategic Planning Division as an advisory team to support the secretariats in their roles:

*‘The Strategic Planning Division can act as mentors for the secretariat, providing motivation, follow-ups, communication, and feedback to clarify roles, address challenges, and monitor indicators.’* (I5)

In addition, external experts could enhance the secretariats’ capacity through strategic administration training:

*‘The Bureau of Tobacco Control should build the secretariat’s capacity based on their needs. There should be external academic consultants who can provide strategic advice. This would enable the secretariat to define clear directions for their work and propose policy recommendations to the chairpersons of each strategy.’* (I2)

*Improve monitoring and evaluation frameworks*

Strengthening the NTCS monitoring and evaluation framework requires clearer indicators, consistent reporting systems, and defined timelines. Currently, indicators are often vague or difficult to measure, and reporting mechanisms vary across agencies:

*‘We have less operational data, like smoke-free markets. Switching from percentage-based indicators to targeted numbers would make evaluations feasible.’* (I9)

Clear timeframes for data collection and evaluations are crucial. Short- and long-term goals ensure consistent tracking:

*‘Each strategy needs roadmaps with quarterly plans and meetings to review progress, challenges, and accomplishments.’* (I10)

At the policy level, the Bureau of Tobacco Control should introduce integrated systems for collecting both process and outcome data. Online tools like Google Sheets and standardized templates can enhance consistency:

*‘The Bureau should create an online reporting template to track budget use and performance indicators.’* (I5)

At the operational level, a hybrid approach using both field visits and real-time digital tools would strengthen alignment with national indicators:

*‘Field visits verify progress, while online platforms like Google Forms provide real-time updates, ensuring alignment with national indicators.’* (I11)

*Enhance policy communication strategies to foster engagement and understanding*

Effective policy communication is vital for promoting understanding and engagement among stakeholders. Reliance on one-way communication has caused misunderstandings and disengagement across inter-ministerial and operational networks. Proactive and interactive strategies are essential to address these challenges:

*‘We should arrange workshops or on-site meetings to explain to ministry leaders why they need to act, show the benefits, and ensure clarity. We must meet them at their convenience; we cannot always expect them to come to us.’* (I4)

Stepwise communication through the Office of Disease Prevention and Control can help bridge national and provincial levels. Regional workshops also provide opportunities for participation and feedback:

*‘The Bureau of Tobacco Control should hold on-site meetings to ensure the Office of Disease Prevention and Control understand the strategic plan and their roles so they can convey this information to operational agencies. Inviting stakeholders to regional workshops would increase involvement, if some cannot attend, virtual meetings can be used.’* (I11)

Additionally, the Bureau of Tobacco Control should also develop videos, summary slides, and concise documents to facilitate understanding. These materials can be shared and adapted by stakeholders for internal communication.

*Develop budget allocation strategies based on local needs*

To improve the effectiveness of NTCS implementation, budgeting practices must be adapted to local needs. The current centralized model fails to account for regional variations in tobacco control challenges. Decentralizing budget management – by allowing the Office of Disease Prevention and Control to manage funds directly to Provincial Public Health Offices – could improve responsiveness and flexibility:

*‘The Office of Disease Prevention and Control should manage budgets within the regions to address actual work demands, while the National Committee for Tobacco Control provides guidance.’* (I12)

A more responsive funding approach should include population health indicators, performance metrics, and innovation potential:

*‘The current criteria, based only on district numbers, are insufficient. We need to consider area size, population, prevalence rates, and administrative accountability.’* (I13)

Additionally, insufficient personnel for tobacco control at the provincial level remains a challenge. Hiring project coordinators would alleviate workloads and improve efficiency:

*‘Coordinators helped with financial documentation and arrangements, significantly streamlining operations.’* (I14)

*Strengthen engagement of government and civil society*

Mobilizing operational networks is essential due to limited support from government agencies. Encouraging governors and provincial committees to formally declare tobacco control as a local priority can help unify stakeholder efforts:

*‘Provincial Tobacco Products Control Committees, chaired by governors, are still new. Inviting governors to declare tobacco control as a priority would help agencies recognize its importance and work towards shared goals.’* (I1)

Several provinces have successfully engaged civil society in campaigns such as alcohol-free festivals. Informants recommended that ThaiHealth should distribute funds to establish a model province for tobacco control by strengthening civil society networks:

*‘We could adopt the alcohol-free network model. It's well-known among communities. If we use a similar approach for tobacco, it could be very effective. ThaiHealth could provide seed funding for forming groups and organizing campaigns.’* (I15)

Local media partnerships also play a vital role in raising awareness and shaping public attitudes:

*‘Regional media networks can advocate and raise awareness. Engaging and training them could make them vital allies in tobacco control.’* (I11)

*Develop a comprehensive database system for planning and evaluation*

Developing a comprehensive and standardized database system is essential to support evidence-based planning and evaluation:

*‘We need data that reflects tobacco consumption, its impact on poverty, and how children become addicted. Common indicators and standardized forms would enable effective comparison and analysis.’* (I3)

It was recommended that regular provincial data collection aligned with NTCS indicators and neutral organizations or academic institutions could take the lead in managing this process:

*‘If we had an annual database, provinces could design solutions to address specific problems. Regional agencies or academic institutions should collect baseline data annually, enabling ongoing surveillance and expansion for future years.’* (I12)

*Facilitate technology and knowledge sharing on successful innovations*

Limited access to up-to-date knowledge, technologies, and innovative approaches has constrained the ability of Provincial Public Health Offices to modernize their tobacco control efforts. Informants emphasized the importance of integrating evidence-based innovations into key strategic areas, particularly youth smoking prevention, smoke-free environments, and cessation services. To address this gap, the Tobacco Control Research and Knowledge Management Center (TRC) should take the lead in synthesizing international evidence and sharing proven innovations with provincial stakeholders:

*‘TRC could review innovations proven effective internationally, such as school programs to prevent e-cigarette use, smoke-free campaigns, and cessation support programs. Findings should be compiled and shared to provide ready-to-use options.’* (I2)

## DISCUSSION

The findings of this study underscore critical challenges and provide actionable suggestions for improving the implementation of Thailand’s NTCS. The challenges and recommendations are applicable to both national and operational levels, and they identify deficiencies in two critical areas: 1) strategic and operational coordination to prioritize tobacco control; and 2) monitoring, evaluation, and reporting systems. Challenges and suggestions specific to the national level include: 3) tobacco control focal point, and 4) policy translation. Challenges and suggestions at the operational level include: 5) budget allocation, and 6) innovation and technology for tobacco control. Addressing these issues requires a systematic and multi-sectoral approach that aligns with global best practices in policy implementation^8^.

### Strategic and operational coordination to prioritize tobacco control

The results highlight the need to foster robust cooperation and networks among agencies at both national and operational levels. At the national level, crucial collaborations involve executives from ministries responsible for the six NTCSs, while at the operational level, engagement with the private sector, civil society, academic institutions, and government agencies is imperative. Such partnerships align with the principles of the FCTC, which advocates for a whole-of-government approach^7,15^. Horizontal coordination among health and other policy domains, together with vertical coordination between national and subnational governments, guarantees collective accountability and dedication to tobacco control initiatives^8,15^. Engagement of stakeholders cultivates a mutual comprehension and joint ownership, which are crucial for prioritizing tobacco control^17,29^. The establishment of institutionalized mechanisms for intersectoral collaboration should be the primary focus of efforts to maintain prioritization and advance the implementation of tobacco control policies.

### Monitoring, evaluation, and reporting systems

The ambiguous indicators and uncoordinated data collection processes in monitoring, evaluation, and reporting systems impedes the ability to effectively evaluate policy performance. This is in accordance with the results that emphasize the importance of integrated reporting systems and precise key performance indicators for the evaluation of tobacco control initiatives^25,29^. The NTCS requires a comprehensive monitoring and evaluation framework that explicitly delineates three critical areas: implementation progress tracking, resource monitoring, and impact assessment^8^. This framework should explicitly identify the specific entities and personnel responsible for data acquisition, analysis, and the synthesis of significant findings and recommendations^8^. The monitoring and evaluation timeline should be strategically aligned with existing surveillance systems to ensure efficient data collection and avoid duplication of efforts^8^. Continuous development and the effective implementation of a tobacco control strategy could be facilitated by the establishment of a synchronized reporting system, specific timeframes, and explicit assessment criteria and accountability mechanisms.

### Tobacco control focal point

The implementation of the FCTC requires a designated ‘tobacco control focal point’ within governmental frameworks^8^. In Thailand, this function is executed by the Bureau of Tobacco Control within the Department of Disease Control, which acts as the secretariat for the SC-NTCS. Nevertheless, high turnover rates and inadequate support systems have considerably undermined the secretariat’s efficacy in executing its vital functions^25^. The secretariat serves as a crucial coordinator at both the policy and operational levels, facilitating communication among ministries and assuring advancement towards NTCS indicators. The dual responsibility, together with its role as the FCTC focal point, requires strong capacity-building initiatives and extensive support mechanisms, including strategic management consultations and ongoing professional development^8^. Enhancing these support systems would bolster the secretariat’s capacity and stability to operate effectively as Thailand’s tobacco control focal point, assuring organizational resilience and continuity in implementation.

### Policy translation

This study’s findings indicate that one-way communication in the dissemination of strategic plans and key performance indicators restricts mutual comprehension and engagement within networks. Successful policy translation necessitates the implementation of a two-way communication paradigm, where stakeholders actively engage in the planning phases and attain a clear understanding of strategic objectives. Workshops, whether held on-site or through online meetings, along with multimedia tools, can improve participation and alignment among stakeholders. These findings are consistent with existing research indicating that two-way communication facilitates active engagement from both policymakers and stakeholders, guaranteeing that policy translation aligns with the requirements and viewpoints of all parties involved^30^. This method conforms to optimal practices in policy translation and execution, highlighting the importance of effective communication and participatory processes to ensure stakeholder engagement^8,31^. Prior research indicates that the efficacy of bidirectional communication in conveying tobacco control policies is contingent upon cultural values, public comprehension of tobacco-related hazards, the political and economic context, and the strategic formulation of messages that align with the priorities and identities of various stakeholders^30^. Future strategies should emphasize inclusive and proactive communication mechanisms tailored to stakeholders’ contexts to foster a shared vision and strengthen policy engagement at all levels.

### Budget allocation

Centralized budget management poses considerable difficulties in meeting local demands and situations. Adopting a decentralized, bottom-up funding approach would allow operational agencies to address region-specific tobacco control concerns more effectively. This approach corresponds with findings from other studies, where decentralized resource distribution has enhanced responsiveness and accountability in public health initiatives^32^. Subnational or local authorities are usually best positioned to implement and enforce tobacco control policies, whereas central administration often leads to inconsistent and suboptimal outcomes^8^. Furthermore, establishing clear criteria for budget distribution and enabling local agencies to manage resources will ensure that financial planning is better aligned with on-the-ground realities^33^. Policy reforms should promote decentralized funding models with transparent criteria to empower local authorities, enhance accountability, and align resources with regional needs, thereby strengthening tobacco control efforts.

### Innovation and technology for tobacco control

Limited use of innovative strategies in tobacco control, especially for adolescent prevention, smoke-free environments, and cessation support, diminishes the effectiveness of existing interventions. A prior study highlighted that innovative strategy utilizing digital tools and behavioral insights have demonstrated potential in decreasing worldwide tobacco consumption^34^. Additionally, academic institutions and research institutes should play a pivotal role in disseminating proven innovations and technology to operational agencies. Sharing successful models can inspire tailored solutions and encourage the adoption of evidence-based practices.

### A comparison with other countries

The findings of this study are consistent with those reported in other countries. In Kenya, key facilitators of tobacco control policy implementation included governmental commitment, stakeholder engagement, and financial support, while major challenges involved limited inter-agency coordination and insufficient enforcement mechanisms^17^. Nigeria’s implementation of its National Tobacco Control Act encountered similar barriers, including weak enforcement capacity and limited governmental commitment^18^. In Egypt and Iran, limited political commitment and insufficient resources for implementation were identified as major difficulties to effective tobacco control policy^35^. Studies conducted in Bangladesh, Ethiopia, India, and Uganda further confirm that coordinating whole-of-government approaches remains a persistent challenge. Identified obstacles include unclear role definitions among agencies, limited inter-ministerial communication, and coordination difficulties among stakeholders^15^. Another study in South Africa and Togo highlighted the need for collaboration and effective communication among government bodies, private sector actors, and civil society organizations to strengthen intersectoral coordination in tobacco control efforts^31^. Additionally, a review of tobacco control policy implementation in low- and middle-income countries emphasized the importance of high-level political commitment, cross-sectoral collaboration, and the active involvement of advocacy groups in supporting policy implementation^36^.

### Strengths and limitations

Despite efforts to recruit informants with substantial experience and primary roles in NTCS implementation, informants were predominantly from the Ministry of Public Health. The study did not capture perspectives from stakeholders in other ministries or sectors, such as private enterprises, and other relevant stakeholders. To enhance the validity of the findings, several measures were taken to minimize bias, including researcher triangulation, reflexive team discussions, and member checking. The interview guide was reviewed by qualitative experts and pre-tested to ensure clarity and relevance. Together, these strategies contributed to the overall trustworthiness of the results. Additionally, although this study provides comprehensive qualitative insights into NTCS implementation at both policy and operational levels within the national context, the findings may have limited generalizability to countries with different socio-political and institutional frameworks from Thailand.

## CONCLUSIONS

This study identifies critical challenges and provides targeted recommendations to improve the implementation of Thailand’s NTCS. First, strengthening strategic and operational coordination through robust intersectoral partnerships is vital for elevating tobacco control as a national priority. Second, establishing effective monitoring, evaluation, and reporting systems with clear criteria and timelines is essential to track progress and ensure accountability. Third, enhancing the capacity of the tobacco control focal point requires robust support systems to address organizational and role clarity deficiencies. Fourth, effective policy translation necessitates adopting two-way communication models to engage stakeholders and foster a shared vision. Fifth, decentralized funding management with transparent criteria can empower local authorities and align resources with regional needs. Finally, fostering innovation and knowledge-sharing mechanisms will enable tailored and scalable interventions that address complex tobacco control challenges. Addressing these areas systematically will strengthen Thailand’s NTCS implementation. Future research should evaluate the impact of these recommendations using mixed-methods approaches that examine both outcomes and the practical application of strategies within policy and health system contexts. Additionally, cross-country comparative studies and international collaborations, particularly with countries implementing similar tobacco control strategies, may provide valuable insights and support the development of scalable solutions tailored to diverse contexts.

## Supplementary Material



## Data Availability

The data supporting this research are available from the authors on reasonable request.
